# When the sun never sets: diverse activity rhythms under continuous daylight in free-living arctic-breeding birds

**DOI:** 10.1098/rspb.2013.1016

**Published:** 2013-08-07

**Authors:** Silke S. Steiger, Mihai Valcu, Kamiel Spoelstra, Barbara Helm, Martin Wikelski, Bart Kempenaers

**Affiliations:** 1 Department Behavioural Ecology and Evolutionary Genetics, Max Planck Institute for Ornithology, 82319 Seewiesen, Germany; 2 Department of Biology, University of Konstanz, 78464 Konstanz, Germany; 3 Department of Migration and Immunoecology, Max Planck Institute for Ornithology, 78315 Radolfzell, Germany; 4 Department of Ecology and Evolutionary Biology, Princeton University, Guyot Hall 403, Princeton, NJ 08540, USA

**Keywords:** circadian clock, continuous daylight, mating system, arrhythmic activity, Arctic, *Calidris*

## Abstract

Circadian clocks are centrally involved in the regulation of daily behavioural and physiological processes. These clocks are synchronized to the 24 h day by external cues (*Zeitgeber*), the most important of which is the light–dark cycle. In polar environments, however, the strength of the *Zeitgeber* is greatly reduced around the summer and winter solstices (continuous daylight or continuous darkness). How animals time their behaviour under such conditions has rarely been studied in the wild. Using a radio-telemetry-based system, we investigated daily activity rhythms under continuous daylight in Barrow, Alaska, throughout the breeding season in four bird species that differ in mating system and parental behaviour. We found substantial diversity in daily activity rhythms depending on species, sex and breeding stage. Individuals exhibited either robust, entrained 24 h activity cycles, were continuously active (arrhythmic) or showed ‘free-running’ activity cycles. In semipalmated sandpipers, a shorebird with biparental incubation, we show that the free-running rhythm is synchronized between pair mates. The diversity of diel time-keeping under continuous daylight emphasizes the plasticity of the circadian system, and the importance of the social and life-history context. Our results support the idea that circadian behaviour can be adaptively modified to enable species-specific time-keeping under polar conditions.

## 1. Introduction

It's a cruel season that makes you get ready for bed while it's light out.

Bill Watterson, Calvin and Hobbes, 1995

Correct timing is essential in many biological processes. To time daily behavioural and physiological processes, most organisms use an endogenous circadian clock, which is synchronized to the 24 h day by one or more external timing cues, known as *Zeitgebers* [1–3]. The most powerful *Zeitgeber* is the daily light–dark cycle [3], to which most organisms are constantly entrained. However, in polar environments, the strength of this *Zeitgeber* is greatly reduced around the summer and winter solstices when the sun never sets (hereafter referred to as ‘continuous daylight’) or never rises. How do animals keep time under weak environmental rhythmicity? Polar organisms could (i) become arrhythmic, (ii) entrain to weaker *Zeitgebers* (e.g. diel changes in light intensity, polarization patterns or sun azimuth [4,5]) or (iii) rely on endogenous rhythms so that they ‘free-run’ with respect to the 24 h day. Only a few studies have investigated activity patterns under polar conditions in the wild and the existing findings are inconsistent [6–14]. In some polar residents, a seasonal absence or reduction of behavioural rhythmicity has been observed [6–8], but in various other species, rhythmicity persisted [9–13], or a free-running rhythm was observed [15–19]. In the arctic ground squirrel *Urocitellus parryii*, body temperatures were arrhythmic during hibernation, but showed robust 24 h cycles after emergence from the hibernacula and throughout the arctic summer [20,21]. In the autumn, after entering the hibernacula, circadian rhythms were free-running as long as the animals were euthermic [20,21].

The diversity of behavioural responses, even within the limited number of species tested, is surprising and suggests that a variety of factors may be involved in regulating circadian plasticity. It has been proposed that circadian clocks can be adaptively modified to enable species-specific time-keeping under polar conditions [6,12,22–24]. Thereby, clocks would be ‘fitted’ to specific aspects of the ecology and behaviour of an organism. For example, it has been hypothesized that for resident herbivores, the evident weakness of the biological clock is an adaptation that enables the animals to feed around the clock [24]. By contrast, insectivorous migrant birds showed continued rhythmicity of long activity bouts during the day and short rest phases at night [9,11,25,26], coinciding with activity and eclosion times of insects [10,13]. Adaptive modification of the circadian system was further supported by parallel findings on diel profiles of melatonin, the most important hormone involved in the regulation of the avian circadian system [12,27–29]. Although plausible, species-specific diel patterns have not yet been rigorously tested by comparative data from free-living animals.

Owing to earlier technical limitations of observing behaviour of wild animals over long time periods, most studies have investigated activity–rest rhythms in captive animals, often under laboratory conditions. However, there is increasing evidence that activity patterns described in captive animals (e.g. wheel running in hamsters) can differ substantially from those of wild animals [30,31].

In this study, we recorded activity–rest cycles in free-living birds belonging to four species that sympatrically and simultaneously breed in similar tundra habitat. We used a standardized, non-invasive, radio-telemetry-based method (see also [32]) that allowed us to follow individuals over several weeks on the arctic breeding grounds. Aiming to substantiate ideas that circadian clocks in the Arctic have evolved in response to the animals' life history, we chose species that represent diversity in mating systems and parental care patterns (summarized in table 1).

**Table 1.** Overview of activity rhythms in four free-living arctic-breeding birds in relation to sex and breeding stage. Activity patterns were classified into three categories based on Lomb–Scargle periodogram analysis: arrhythmic, no periodicity could be detected; entrained, a significant periodicity was detected that did not differ from 24 h; free-running, a significant periodicity was detected that deviated significantly from 24 h. Note that the three shorebird species have precocial young (parental care only includes brooding, attending and defending the young), whereas the Lapland longspur has altricial young (fed by both parents).

species	social mating system^a^	parental care pattern^a^	sex	activity pattern		
				arrhythmic	entrained	free-running
semipalmated sandpiper	monogamous	biparental	male	pre-incubation	—	incubation
			female	pre-incubation	—	incubation
pectoral sandpiper	polygynous	female only	male	entire season	—	—
			female	pre-incubation	incubation	—
red phalarope	polyandrous, sex-role reversed	male only	male	pre-incubation	incubation	—
			female	entire season	—	—
Lapland longspur	monogamous, occasional polygyny	biparental (female-only incubation)	male	—	entire season	—
			female	—	entire season	—

^a^Sources: [33–36].

We examined the timing of activity of males and females in four long-distance migrants: three closely related shorebirds (family Scolopacidae) and one songbird (family Emberizidae). Shorebirds are particularly attractive for chronobiological studies, because diverse activity patterns have been observed but rarely studied in detail [29,37–39]. Songbirds provide a useful comparison because their circadian systems have been studied extensively [27,28,40]. In the two polygamous shorebirds and the socially monogamous songbird, only one sex incubates, whereas incubation is shared in the semipalmated sandpiper, a monogamous shorebird species. In the two polygamous shorebirds, the non-incubating sex predominantly competes for mating opportunities. We generally hypothesized that seasonal constraints and variation in life histories will affect activity rhythms. Specifically, the narrow annual window of opportunity for breeding, combined with continuous light availability during the short arctic summer, should heighten pressure on birds to adjust activity patterns for maximal reproductive benefits (e.g. increase the duration of activity to pursue additional mating opportunities, or time activity in order to maximize foraging efficiency). Hence, the different mating systems and parental care patterns should relate to different selective advantages of maintaining internal clocks under almost constant light conditions. If so, activity rhythms should be species- and sex-specific, reflecting not only foraging opportunities, but also mating opportunities and parental responsibilities. A recent study on birds reported that circadian period length is heritable and suggested that properties of circadian clocks can affect performance in sexually selected traits [41]. Hence, the aim of this study is to describe the activity rhythms of the four arctic-breeding species, and assess variation in relation to mating system, sex and breeding stage.

In addition, we assessed the amplitude of the circadian system in the field by examining diel melatonin profiles in the semipalmated sandpiper because of its biparental care. It has previously been suggested that entrainment to subtle *Zeitgebers* would be facilitated by a low-amplitude melatonin cycle. We therefore tested the idea that in a species where breeding partners need close temporal coordination of activity, melatonin cycles had particularly low amplitudes [38,42].

Our results show that a diversity of activity patterns (24 h entrained rhythm, ‘free-running’-like rhythm and arrhythmicity) can occur under the same environmental conditions, depending on the life history of the species, the sex of the individual and the breeding stage. This indicates that the circadian system may be more plastic than previously thought, and that arrhythmicity is not exclusively found in polar residents. Furthermore, plasma melatonin rhythmicity was undetectable in semipalmated sandpipers, in contrast to melatonin profiles reported for arctic songbirds [12,27] and for shorebirds kept under natural central European day length conditions [29]. Our study highlights the value of studying arctic animals for understanding timing strategies and for testing the malleability of the circadian system under natural conditions.

## 2. Methods

### (a) General field procedures and radio-transmitter use

**Figure 1.**
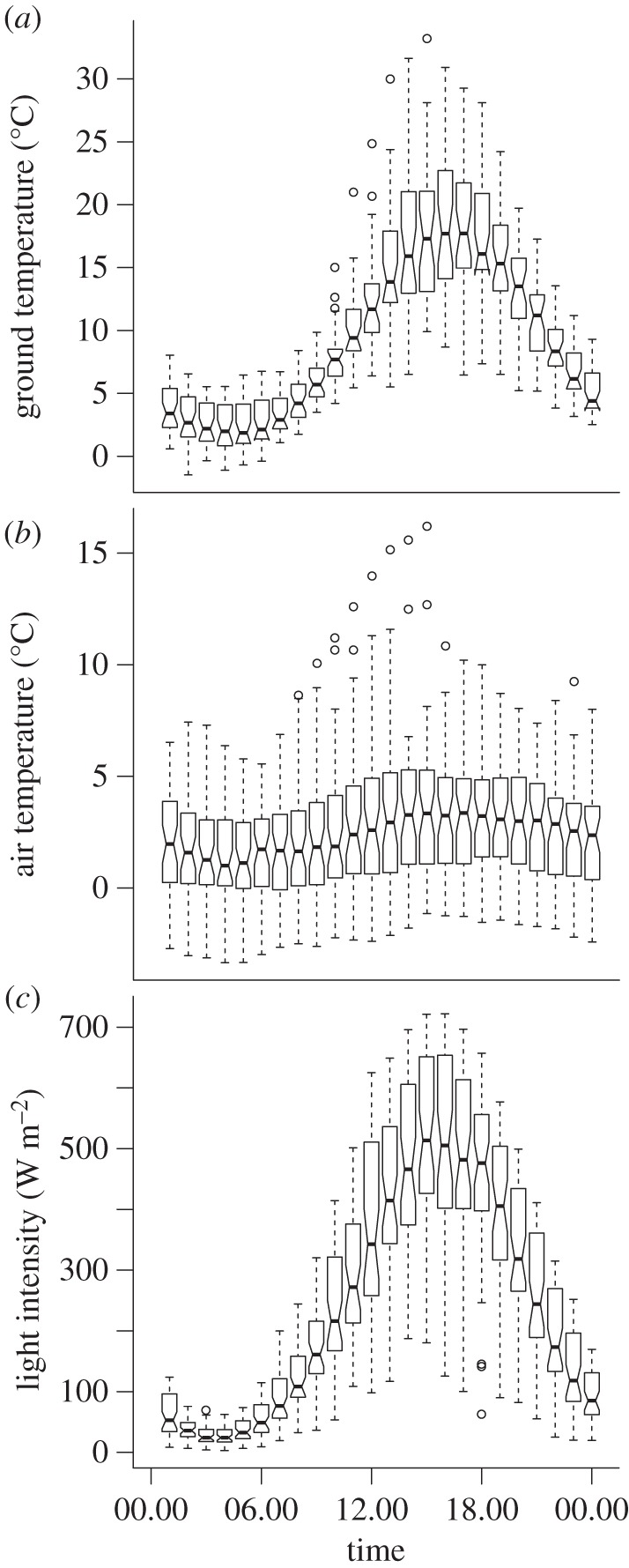
Daily environmental cycles in Barrow, Alaska. (*a*) Ground temperature (°C). (*b*) Air temperature (°C). (*c*) Light intensity (W m^−2^). Shown are box plots with hourly median, 25th and 75th percentile, 9th and 91st percentile, and outliers. Ground temperature data were recorded between 14 June and 18 July 2006 near 27 nests of pectoral sandpipers using iButtons (accuracy ± 1°C). Air temperature (2 m above ground) and light intensity data were collected by weather stations located in Barrow from 1 June to 20 July 2008. These data were kindly provided by the NOAA Earth System Research Laboratory (http://www.esrl.noaa.gov). Note that light intensity was measured with an unshaded pyranometer (results referred to as ‘global’), and may yield erroneous results at extremes of zenith angle (R. Stone 2012, personal communication). Near-zero values can occur at low solar elevation in cloudy conditions.

We studied semipalmated (SESA) and pectoral sandpipers (PESA), red phalaropes (REPH) and Lapland longspurs (LALO) in June–July 2007–2008 in a 2 km^2^ study area near Barrow, Alaska (71°32′ N, 156°65′ W); (for more details on the study site, see [14,43]). The site consists of wet coastal–plain tundra vegetation. The birds arrive in late May to mid-June and experience continuous light throughout their breeding season. Despite continuous daylight, ambient temperatures and light intensity varied on a 24 h basis (figure 1). We captured birds with mistnets or in walk-in traps on the nest between 4 June and 13 July. From each individual, we sampled blood (50–200 µl), measured wing, tarsus, culmen and total head length (to the nearest mm), and took the weight (to the nearest g). Each bird was banded with a unique combination of colour bands, a green flag (shorebirds only) and an aluminium band from the Bird Banding Laboratory of the US Geological Survey Patuxent Wildlife Research Center. We determined sex based on morphology, behaviour and molecular markers [44,45].

We searched nests throughout the breeding season by observing the behaviour of birds, either when flushed during laying or incubation, or on incubation breaks, and monitored their fate. If shorebird nests were found after clutch completion, then egg development was assessed through flotation [46]. The start of the incubation period was estimated as date of hatch minus 22, 20, 19 and 12 days for PESA, SESA, REPH and LALO, respectively.

We equipped 142 birds (21 LALO, 42 PESA, 47 REPH, 32 SESA) with glue-on, light-weight (less than 5% of body mass) radio-transmitters (2007: BD-2, 1.05 g, Holohil Systems Ltd, Canada; 2008: A2420, 1.3 g, Advanced Telemetry Systems, USA) with a frequency range of 164–167 MHz. After release, all radio-tagged birds behaved normally.

### (b) Automated activity recording and behavioural observations

We used two to four automated receiving units (ARUs), each connected to a directional four-element Yagi-antenna and powered by a car battery, to simultaneously and continuously monitor the activity of the free-living, radio-tagged birds [32,47]. The ARUs scanned through all programmed frequencies (deployed transmitters) in 1 min intervals, and recorded background noise and signal strength (in dB). Radio-signals were detected up to distances of approximately 1 km. Raw data were saved in receiver memory, and subsequently saved on a PC for further analysis.

We applied a minimum signal threshold of −129.89 to −119.87 dB, depending on the ARU and antenna set-up, above which the signal exceeded background noise. Signal strength did not change much when an animal (i.e. the transmitter) was stationary, but varied when the animal was moving or changed posture. Therefore, we used the change (Δ) in signal strength between subsequent 1 min intervals to quantify whether an animal was active or not. We determined threshold values of Δ signal strength to distinguish between activity and inactivity through field calibration by direct observation of the birds' activity. Using synchronized clocks, we monitored 36 tagged birds (nine LALO, six PESA, six REPH, 15 SESA; both sexes) for 1.5–6 h (average: 130 ± 17 min) and at 1 min intervals recorded behaviour (active: foraging, flying, preening, fighting, displaying; inactive: sitting, resting, sleeping, incubating).

We analysed Δ signal strength data with a general linear mixed-effect model (GLMM) with binomial error distribution. Models included activity (1/0; as determined from behavioural observations) as dependent variable, species and sex as fixed factors, and individual as random factor. Δ signal strength values differed significantly between active and inactive birds (see the electronic supplementary material, figure S1; GLMM, *F*_1,1762_ = 32.94, *p* < 0.001) but not between species (*F*_3,32_ = 1.56, *p* = 0.21). Based on this and similar analyses of data from other species (www.sparrowsystems.biz, ARTS activity manual), we used a Δ signal strength of 3.8 dB as threshold value to characterize an animal as ‘active’ (Δ ≥ 3.8 dB) or ‘inactive’ (Δ < 3.8 dB). This value equals the upper end of the 99% CI of the mean for inactive animals. Note that using thresholds of 3 and 5 dB gave qualitatively similar results. Missing data points (i.e. values below the background noise level) were caused, for example, by animals venturing outside the system's recording range, by brief system shutdown for data retrieval, by the antenna of the radiotransmitter being under water (e.g. red phalaropes are often found on water), or by the antenna being in contact with the ground or masked by the micro-relief features. The mean number of recordings per individual within a 5 min interval was 2.62 (95% CI: 2.52, 2.73). The mean number of recordings did not differ between species (likelihood ratio test of a mixed-effect model with ID as random intercept, *χ*² = 3.27, d.f. = 3, *p* = 0.35; number of observations: 340 980; number of individuals: 117).

### (c) Melatonin measurements

In 2004, we captured 108 SESAs during the breeding season at different times throughout the 24 h cycle (see figure S2 in the electronic supplementary material for detailed sampling regime), either with walk-in traps on the nest (*n* = 78) or with mistnets (*n* = 30). Thirteen individuals were caught and bled twice, on different days (mean interval: 10 ± 1.8 days, range: 3–21 days) and at different times.

Blood samples were centrifuged for 10 min at 1500*g* to separate the plasma, which was stored immediately at –20°C for subsequent analysis. Plasma samples (40–100 µl) were transported to Germany on dry ice and kept at −80°C. Melatonin was assayed in duplicates by radioimmunoassay after extraction on diatomaceous earth columns (for details, see [48]). We conducted three extractions and two assays. Samples were arranged so that every extraction and assay contained samples from each 2 h interval. Average detection limit was 18.9 ± 0.1 pg ml^−1^, average recovery was 50.1 ± 9.7 per cent (recovery extraction 1 = 69%; recovery extraction 2 = 42%; recovery extraction 3 = 39%), intra-assay variation was 3.7 ± 2.2 per cent and inter-assay variation was 4.3 per cent.

### (d) Data analysis and statistics

We performed all analyses with the R v. 2.9.0 software [49]. For rhythmic analysis of activity patterns, we created time series from the ARU as described above. These continuous activity records were used to assess significant circadian periodicity and to calculate period length using the Lomb–Scargle periodogram analysis implemented in ChronoShop (software developed by K.S. can be downloaded from https://www.nioo.knaw.nl/users/kspoelstra). This method can detect periodic components in datasets with missing values, and is therefore ideally suited for telemetry-based time series obtained from free-living animals [50]. The analyses were performed for periodicities ranging from 10 to 30 h with 4 min increments. The program calculates a Lomb–Scargle PN value, which is the normalized power as a function of angular frequency (*ω* = 2*π*/*P*) for all periods (*P*) tested, and tests whether this value exceeds the significance threshold. Period length was considered significantly different from 24 h if the average plus or minus the standard error did not overlap with 24 h.

For the analyses of activity cycles, we distinguished between three breeding stages: pre-incubation (before completion of the clutch), incubation (after clutch completion, before hatching) and post-incubation (after hatching). We conducted periodogram analysis only for individuals with more than 6 days of continuous activity records per breeding stage. We used the results of the periodogram analyses to distinguish between arrhythmic (i.e. non-significant periodicity), entrained (i.e. significant periodicity near 24 h) and free-running (i.e. significant periodicity deviating from 24 h) activity rhythms (table 1). Free-running birds typically showed fast cycles at first, followed by a marked increase in period length. In these birds, we estimated period length separately for a free-run before and after a change in direction (i.e. from period length shorter than 24 h to those longer than 24 h). Because we collected few data from birds during the post-incubation period, this period was excluded for detailed analysis.

## 3. Results

**Figure 2.**
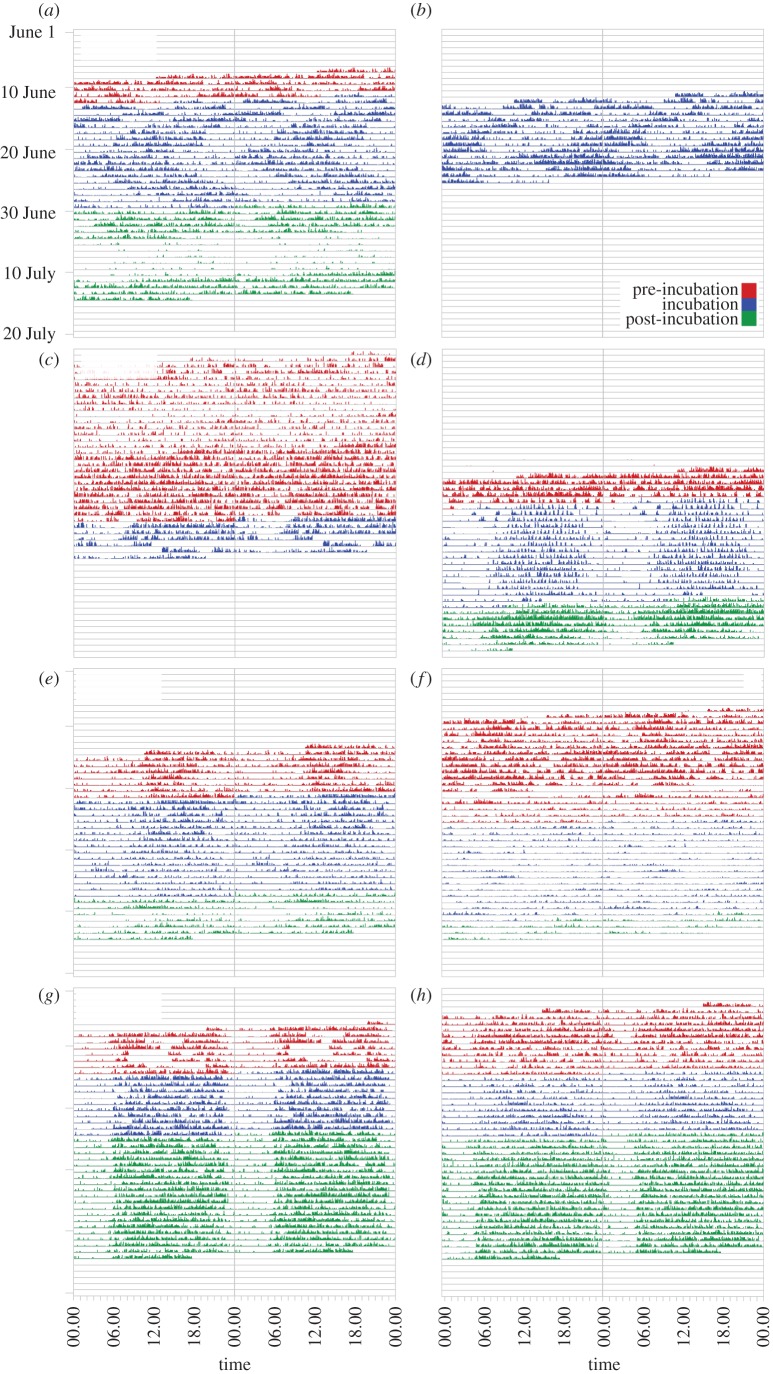
Actograms of males and females from four bird species breeding in Barrow, Alaska. (*a*) Male and (*b*) female semipalmated sandpiper *Calidris pusilla* (SESA) from the same nest. (*c*) Male and (*d*) female pectoral sandpiper *Calidris melanotos* (PESA). (*e*) Male and (*f*) female red phalarope *Phalaropus fulicarius* (REPH). (*g*) Male and (*h*) female Lapland longspur *Calcarius lapponicus* (LALO). Each actogram shows the activity records of one individual over a 24 h period, plotted twice, such that each row represents two consecutive days. Coloured regions indicate activity, whereby bar height is proportional to the amount of activity within a 5 min interval. Colour indicates breeding stage: pre-incubation (red), incubation (blue), post-incubation (green). Note that for male PESA and female REPH, breeding stage refers to the population breeding stage (i.e. 95% of individuals in the population incubating, or having offspring), and for male LALO it refers to the breeding stage of its mate (males do not incubate, but they do provision nestlings). Continuous activity data are only available for a few days for female REPH, because they left the male after the clutch was completed.

**Figure 3.**
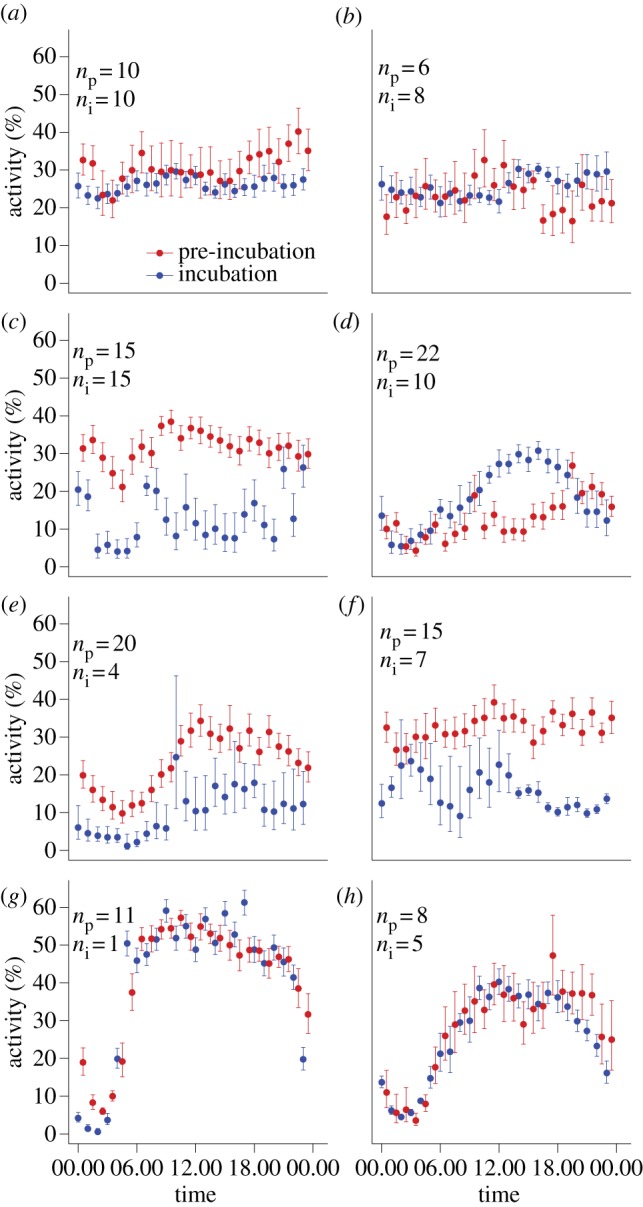
Mean hourly activity for males and females from four bird species breeding in Barrow, Alaska. (*a*) Male and (*b*) female semipalmated sandpiper *Calidris pusilla* (SESA). (*c*) Male and (*d*) female pectoral sandpiper *Calidris melanotos* (PESA). (*e*) Male and (*f*) female red phalarope *Phalaropus fulicarius* (REPH). (*g*) Male and (*h*) female Lapland longspur *Calcarius lapponicus* (LALO). Data were collected between 2 June and 19 July 2007, and between 3 June and 14 July 2008. Shown are the mean ± s.e. of hourly activity estimated as the intercept of a mixed-effect model with binomial error distribution where activity (yes/no) was the dependent variable and individual ID was included as random factor. Estimates were back-transformed to the original scale. For male PESA and female REPH, the incubation stage refers to the period when 95% of individuals (of the opposite sex) in the population were incubating. For male LALO, incubation stage refers to the period when the female mate was incubating.

Actograms revealed substantial variation in daily activity rhythms, depending on the species and sex (figure 2). In the three sandpiper species, marked seasonal changes in daily activity patterns occurred even within individuals, in particular in association with the onset of incubation (figures 2 and 3, table 1).

The periodogram analysis clearly shows the absence of rhythmic activity patterns in the non-incubating sexes of the two polygamous species (figure 2*c,f*, table 1). Male pectoral sandpipers (*n* = 9) and female red phalaropes (*n* = 6) were continuously active over the 24 h period, without long rest phases. Lomb–Scargle periodogram analysis did not detect any significant periodicities during the entire recorded presence of these individuals. Similarly, no significant periodicity could be detected for any of the sandpipers in the pre-incubation period (female pectoral sandpipers, *n* = 2; male red phalaropes, *n* = 5; male and female semipalmated sandpiper, *n* = 4 and 1, respectively).

In contrast to the shorebirds, Lapland longspurs generally displayed a typical diurnal pattern of activity, which remained more or less constant throughout the breeding season (figure 2*g*,*h*). Rest times were approximately between midnight and 4.00 (figures 2*g*,*h* and 3*g*,*h*) when light intensity and temperatures were at their lowest (figure 1). Both males and females showed a robust activity rhythm with a 24 h period length (table 2). We also observed a diel rhythm with a 24 h period in the male red phalarope and female pectoral sandpiper, at least during the incubation period (figures 2*d,e* and 3*d,e*, table 2).

**Table 2.** Mean and s.e. of circadian period length (h) in four free-living arctic-breeding birds. Data are based on Lomb–Scargle periodogram analysis. *n* refers to the number of individuals. For the three sandpipers, only data from the incubation period are included, because birds were arrhythmic in the pre-incubation period. For the Lapland longspur, all breeding stages were combined, because patterns did not change seasonally.

species	sex	period length			*n*
		mean	s.e.	range	
semipalmated sandpiper	male	25.90	0.71	21.73–28.60	9
	female	24.68	1.97	21.20–28.80	4
pectoral sandpiper	female	24.05	0.02	24.00–24.07	3
red phalarope	male	24.01	0.08	23.80–24.40	6
Lapland longspur	male	23.98	0.04	23.60–24.20	9
	female	23.96	0.03	23.87–24.07	8

During the incubation period, males and females of the biparental semipalmated sandpiper showed remarkable activity patterns (figure 2*a,b* and table 2). The birds were clearly rhythmic, but period lengths were significantly different from 24 h, suggestive of a free-running rhythm. However, many individuals showed dramatic shifts in period length, and the overall range of periodicities was large (roughly 21–29 h). Thus, activity patterns differed from the consistent rhythmicity expected from endogenous, circadian clocks, and may have arisen from different origins or from weak entrainment. Although period length varied among individuals, there was no difference between the sexes (table 2).

In all samples, melatonin levels were below 18.9 pg ml^−1^ (detection limit), independent of time of day (see the electronic supplementary material, figure S2). Although in two out of three extractions recovery rates were low, we are confident that increased recoveries would not have changed our results. Even in extraction 1 with 69 per cent recovery, plasma melatonin was below the detection limit in all cases. Furthermore, plasma melatonin was not detectable in eight birds sampled during periods of low light intensity (21.00–03.00) despite large plasma volumes (more than 80 µl) and good recoveries (more than 70%).

## 4. Discussion

Despite similar environmental conditions with continuous daylight, we find substantial diversity in daily activity rhythms of free-living birds, depending on species, sex and breeding stage. All species are migratory and—except for the short arctic summer—experience typical light–dark regimes. Individuals exhibited either robust 24 h activity cycles, were continuously active (arrhythmic) or showed activity cycles suggestive of ‘free-running’ circadian rhythms. We now discuss each of these patterns in turn.

### (a) Entrained 24 h rhythms under continuous daylight

Lapland longspurs showed a robust 24 h rhythm of activity during the day and rest during the (short) ‘night’ (figure 2*g,h*). These findings are consistent with observations that rhythmicity persisted under continuous natural daylight in other species, including birds [4,5,9,12,23], arctic ground squirrels [20,42] and bumble-bees [13]. Likewise, melatonin, a hormone that plays an important role in the circadian system, was shown to maintain diel rhythmicity under continuous polar daylight in longspurs [27,28] and in another passerine (willow warbler *Phylloscopus trochilus* [12]). Although plasma melatonin concentrations were drastically reduced compared with those of songbirds at lower latitudes, levels were still elevated at night (see figure S2 in the electronic supplementary material and [12,28]). Together, this suggests that the circadian system of the Lapland longspur remained fully rhythmic and entrained throughout the breeding season under continuous natural daylight conditions. A recent study suggests that daily fluctuations in ambient temperature and in light intensity or quality (figure 1) can act as synchronizers [28].

During the incubation period, we also observed a rhythm with a clear 24 h period in the care-giving sex of the uniparental shorebirds (figure 2*e,d*). These birds were also more active during the day than at night, which is consistent with studies showing higher nest attendance at night [51]. Synchronization to the polar day may be adaptive because of daily temperature fluctuations (figure 1). Diel changes in temperature are substantial at ground level (figure 1*a*), and cause variation in both the need for continuous incubation (eggs cool off faster at ‘night’) and in foraging efficiency (invertebrate food is harder to access at ‘night’ [11,52]).

### (b) Behavioural arrhythmicity and reproductive sleeplessness

Our data show the absence of rhythmic activity patterns in all sandpipers in the pre-incubation period (figure 2*a,c,d–f*), and in the competitive sex of the two polygamous species throughout the season (figure 2*c,f*). A similar seasonal absence or reduction of rhythmicity has been observed in two polar residents: the Svalbard ptarmigan *Lagopus mutus hyperboreus* [6,7] and reindeer *Rangifer tarandus platyrhynchus* [8]. In herbivores, in particular, the evident weakness of the biological clock has been interpreted as an adaptation enabling individuals to feed around the clock [24]. Our data show that a seasonal absence of rhythmicity is not unique to resident polar vertebrates [24]. However, in sandpipers, which feed on invertebrates, arrhythmicity was breeding-stage-specific and sex-role-specific. In the two polygamous species, the incubating sex was rhythmic only during incubation, whereas the non-incubating sex was active around the clock. In early June, ground temperatures often dropped below freezing level during the ‘night’ (figure 1*a*), substantially reducing arthropod availability [52]. This makes foraging around the clock an unlikely explanation for arrhythmicity. Instead, we suggest that around-the-clock activity of arctic shorebirds represents ‘reproductive sleeplessness’ (i.e. around-the-clock competition for access to mates) [14]. In pectoral sandpipers, we showed earlier that a radio-telemetry-based estimate of activity coincides with high electromyogram activity and wakefulness [14], and that males that were the most active sired the most offspring. This suggests that arrhythmicity with nearly constant activity has evolved in a continuous daylight environment in response to intense sexual selection (competition for mates). In general, we would then expect to find arrhythmicity in arctic polygynous or lekking species with a strong mating skew.

### (c) Free-running, sociable rhythms in biparental shorebirds

During the incubation period, male and female semipalmated sandpipers showed activity patterns with variable period lengths that significantly differed from 24 h. Our data provide preliminary evidence for social synchronization among breeding partners that ‘free-ran’ with respect to the 24 h day. For three pairs for which sufficient data were available, period lengths (male–female) were estimated as 27.9–28.8, 27.0–27.3 and 21.7–21.4 h, respectively. This suggests that the daily rhythms of pair members were synchronized with complementary—but not necessarily equal—activity and incubation patterns (figure 2*a,b*).

The basis of this rhythmicity and possible social synchronization is still unclear. Close synchronization with 24 h *Zeitgebers* (figure 1) can be excluded because of the observed range of period lengths. Tidal rhythms can also be excluded because they provide signals that are 24.8 h in length. Consequently, our results suggest that social cues directly related to reproductive activity may act as a *Zeitgeber* which overrules the 24 h *Zeitgebers* and which allows coordination of daily activity rhythms (i.e. incubation bouts). Although there is some evidence for entrainment by conspecifics [53], social cues are generally believed to be a relatively weak *Zeitgeber* and to affect timing mainly in the absence of other, stronger *Zeitgebers*. Thus, continuous daylight in the arctic summer might facilitate synchronization by social *Zeitgebers* [27,40,54]. Furthermore, the more weakly self-sustained a circadian system is, the more readily it is entrained by subtle *Zeitgebers* [29,40]. A recent study on two other wader species reported very low amplitudes in the daily cycle of the circadian hormone melatonin [29]. During the polar summer, melatonin is expected to be further suppressed by continuous daylight. In support of this idea, and in contrast to reports from songbirds [12,27,28], we found evidence that plasma melatonin rhythmicity was undetectable in free-living semipalmated sandpipers. Elevated levels of plasma melatonin were absent in birds caught during day and night (see the electronic supplementary material, figure S2). At present, we cannot rule out that there is a very low amplitude melatonin peak below the detection limit of our assay. We also lack comparative data from other shorebird species to determine whether melatonin was particularly low in the biparental semipalmated sandpiper. Nonetheless, the low amplitude of the birds' melatonin cycle may facilitate entrainment to social cues.

In support of social synchronization, observations of nest attendance [44] (figure 2) and direct observations of changeovers between incubating birds (S.S.T., M.V., K.S., B.H., M.W. & B.K. 2011, 2012, unpublished data) indicate that changeovers at the nest were mostly instantaneous. If mates initially differ in temporal preference, then social synchronization implies adjustment of individual schedules [55]. For example, the dominant bird might impose its own rhythm on the partner, or the observed period length might reflect a value intermediate between the period lengths of the initial rhythms (mutual synchronization [53]). Behavioural observations suggest that the timing of the incubation shift is determined by the incubating bird [43], but this is based on a small sample size. Variation in the length of incubation bouts may also reflect body condition of the parents, differences in the microclimate around the nest, or experience or age of the parents [44,56].

Surprisingly, we observed intra-individual shifts in period length during the incubation period in six out of 11 individuals (figure 2*a,b*). In all such cases, birds shifted from a short (less than 24 h) to a long (more than 24 h) period, and thereby increased overall length of incubation bouts. Thus, a potential interpretation of these observations is that individuals adjusted incubation bout length over the season, for example, owing to improving environmental conditions. Longer incubation bouts may be advantageous to decrease the risk of predation, assuming that nest detection by predators is highest during the changeover. This is not unlikely for the most common avian nest predators, the glaucous gull (*Larus hyperboreus*) and different species of skua (*Stercorarius* sp.), which hunt visually and are very common in the study area. Likewise, incubation bout length may be limited by the need of the incubating bird to replenish its energy reserves [44,57]. As it becomes warmer with the progress of the season, incubation bouts could lengthen, because energetic costs should decline while foraging efficiency increases. Previous work suggested that female semipalmated sandpipers incubate more during the night than males [44]. However, our data contradict this. The observed free-running rhythm implies that for each pair member the timing of the incubation bout shifts over consecutive days, such that neither sex will have to incubate or forage exclusively during the (energetically) worst part of the day. Thus, the fact that these birds do not seem to use weak *Zeitgebers* to synchronize their activity with the 24 h day may be an adaption to biparental incubation.

True free-running rhythms—based on an internal clock—have rarely been observed in nature and are usually much closer in period length to 24 h than the rhythms we detected [15–19]. We propose that the incubation/activity rhythms observed in semipalmated sandpipers are not true free-running rhythms reflecting an individual's internal clock, but rather rhythms that are the outcome of selection on incubation bout length and/or of processes of social negotiation. This suggests that such activity rhythms may be more generally found in biparentally incubating birds that breed under polar light conditions.

## 5. Conclusions

We here provide the first compelling evidence based on behaviour of free-living, arctic-breeding birds for the existence of marked plasticity and substantial diversity in daily activity rhythms among species, between males and females of the same species, and between individuals in different stages of reproduction, from a single site and a single, brief observation period. Our study highlights the diversity of diel time-keeping strategies, and emphasizes the plasticity of the circadian system and the importance of the social context. We provide descriptive data on timing strategies ranging from sociable schedules that ‘free-run’ with respect to the environment, to arrhythmic, continuous activity during the mating season, to shifts to entrained rhythmicity, all at one location during a single, polar summer.

Our results indicate that the circadian system may be more plastic than previously thought [58]. The results show that arrhythmicity is not exclusively found in polar residents, but occurs just as well in migratory species that live in a typical day–night environment outside of the short breeding season. Rather than being strictly determined by residence, our preliminary data suggest that circadian systems are fitted to the social system of a given species, at least under arctic conditions. The remarkable temporal plasticity in waders could be facilitated by the low amplitude of melatonin, in contrast to the situation in songbirds [12,27,28].

We suggest several avenues for further work. First, comparative analyses would allow formal testing of the relationship between daily activity patterns and life-history traits (e.g. mating system, migratory behaviour). To this end, activity data on a larger number of species breeding in polar regions need to be gathered. Second, the adaptive value of various activity patterns is still poorly understood. This can be addressed by studying the fitness consequences of variation in activity patterns observed within species (as in [14]). Finally, the free-running-like rhythm observed in a biparental shorebird suggests that rhythms with periodicities that are close to 24 h could arise from physiological processes other than circadian rhythms, although the mechanistic basis of the observed daily activity cycles remains unknown. Our study underpins the intriguing potential of studying arctic species for an advanced understanding of both animal behaviour and circadian biology. Under released temporal constraints, contributions of specific ecological factors to timing strategies can become evident, and properties of the circadian system, such as evolutionary malleability and its relationship to species biology and social context, come into view.

## Data accessibility

The data can be found at Dryad doi:10.5061/dryad.4g1g0.
